# Emodin Regulates lncRNA XIST/miR-217 Axis to Protect Myocardial Ischemia-Reperfusion Injury

**DOI:** 10.1155/2023/3612814

**Published:** 2023-01-31

**Authors:** Shuai Huang, Lailiang Xue, Qiaona Mou

**Affiliations:** ^1^Shandong Yuncheng Center for Disease Control and Prevention, Heze, 274700 Shandong, China; ^2^Department of Cardiology, Gaoxin District People's Hospital of Linyi, Linyi, 276000 Shandong, China; ^3^Department of Cardiology, Yantai Laiyang Central Hospital, Yantai, 265200 Shandong, China

## Abstract

**Purpose:**

This study is aimed at investigating the effect of emodin on myocardial ischemia-reperfusion injury (MIRI) and mechanism.

**Methods:**

Eighty healthy adult male SD rats (weighing 230-250 g) were utilized to establish I/R model, which were randomly divided into five groups (16 rats in each group): sham operation group, myocardial ischemia-reperfusion injury group (I/R group), emodin group, emodin +NC group, and emodin +XIST group. The contents of CK, CK-MB, LDH, and HBDH in serum were determined by ELISA kit. LDH was detected by ELISA assay, SOD was detected by the xanthine oxidase method, and MDA was detected by the thiobarbituric acid method. The relative expression of XIST and miR-217 was evaluated by RT-qPCR. Western blot was applied to detect the protein expression. Flow cytometry was applied to detect cardiomyocyte apoptosis.

**Results:**

Myocardial infarction area was obviously increased in I/R model rats, while emodin decreased the myocardial infarction in I/R model rats. In addition, cardiac enzymes (CK, CK-MB, LDH, and HBDH) and apoptosis were obviously increased in MIRI model rats, while emodin obviously decreased cardiac enzymes and apoptosis. The ROS and MDA levels were raised while the activities of SOD were declined in the I/R model group. The ROS and MDA levels were decreased while the activities of SOD were raised in the emodin group. The XIST expression was markedly raised in the I/R model group while decreased in the emodin group, and the overexpression of XIST reversed the protective effect of emodin on myocardial infarction, oxidative stress, and cardiomyocyte apoptosis. In addition, XIST directly regulated the expression of miR-217, and si-XIST inhibited H/R-induced oxidative damage of cardiomyocytes via inhibiting miR-217.

**Conclusion:**

Emodin protected MIRI both in vitro and in vivo via inhibiting lncRNA XIST to upregulate miR-217.

## 1. Introduction

Although the current treatment of acute myocardial infarction (AMI) has some effect, AMI and frequent heart failure are still the main causes of death [1]. Therefore, new treatment strategies are needed to protect the heart from the harmful effects of acute ischemia/reperfusion (I/R) injury. Myocardial ischemia-reperfusion injury (MIRI) refers to a series of reactions such as enlargement of infarct area and low cardiac function after successful recovery of cardiac blood flow, which not only fails to restore its function but also aggravates the myocardial injury. A large number of studies have shown that drug preconditioning has a certain protective effect on myocardial ischemia/reperfusion injury. In contract to drugs with broad-spectrum mechanism of action, drugs specifically targeting mitochondrial function have not proved to have obvious clinical significance [2]. China is rich in traditional Chinese medicine resources. It is of great clinical value to find drugs that can effectively protect the heart against ischemia/reperfusion injury. Emodin is an anthraquinone derivative from the rhizome of Rheum palmatum, which is widely used as a laxative in traditional Chinese medicine [3–5]. Emodin significantly reduced the level of inflammatory factors in myocardial injury and relieved LPS-induced myocardial injury [6]. Emodin can alleviate MIRI and has a good anti-inflammatory effect [7].

MIRI is a common pathophysiological manifestation in clinic, and its pathogenesis may be closely related to many factors, such as increased free radical production, metabolic disorder of myocardial fiber capacity, increased nitric oxide, and activation of neutrophils [8]. Relevant research reports have confirmed that reducing cardiomyocyte apoptosis is conducive to improving the prognosis of MIRI. Apoptosis is a way of cell death, that is, programmed cell death. More and more evidence shows that apoptosis is an important pathogenesis of MIRI [9, 10]. In the rat ischemia-reperfusion injury model, it can be found that the apoptosis rate is obviously higher than that in the normal group [11]. Heusch found that ischemia-reperfusion rats have typical DNA breakage and apoptotic body formation; that is, reperfusion injury can have typical apoptotic phenomenon [12]. Activation of reactive oxygen species, intracellular calcium overload, and mitochondrial energy metabolism disorder is the main mechanisms of apoptosis in MIRI [13]. The caspase family plays a major role in the executive stage of apoptosis [14]. Apoptosis is accomplished by a variety of caspases. Caspase-3 is considered to be the terminal shear enzyme that various apoptosis-stimulating factors promote apoptosis [15]. Some studies have shown that caspase-3 activation is the main mechanism of myocardial cell apoptosis leading to the aggravation of myocardial infarction after reperfusion [16, 17]. In the early stage of MIRI and during reperfusion, apoptosis will aggravate myocardial injury. The study of blocking the signal pathway of apoptosis may provide a new theoretical basis for the prevention and treatment of MIRI. However, the effects of emodin on oxidative stress and apoptosis in MIRI and its underlying mechanisms have not been reported.

This study is mainly aimed at exploring the effects and potential mechanisms of emodin on oxidative stress and apoptosis in MIRI.

## 2. Materials and Method

### 2.1. Animals

Healthy adult male SD rats (weighing 230-250 g) were purchased from SBF Biotechnology Co., Ltd. and fed in a sterile and quiet environment with a temperature of 18-26°C and a humidity of 40%-70%. Rats were fed with fresh and clean sterile feed at fixed time every week. All animal experiments were carried out in accordance with the Guidelines for Laboratory Animals of the Animal Experiment. After three days of feeding, they were randomly divided into five groups (16 rats in each group): sham operation group, myocardial ischemia-reperfusion injury group (I/R group), emodin group, emodin +NC group, and emodin +XIST group. The I/R model was constructed. The left anterior descending coronary artery was ligated, and the heart was reset after ligation. After ischemia for 30 min, the ligation line was cut and had reperfusion for 120 min. The heart was quickly cut and placed in ice normal saline. The heart was washed and stored in a refrigerator at -80°C. The sham operation group was operated in the same way without ligation. Rats were injected with emodin 1 hour before I/R treatment.

### 2.2. Cell Line and Transfection

Immortalized rat H9C2 cardiomyocytes were obtained from ATCC and were cultured in DMEM with 10% fetal bovine serum and penicillin/streptomycin (Gibco, USA) at 37°C with 5% CO_2_. To establish a cellular hypoxia-reoxygenation (H/R) model, H9C2 cells were maintained in anaerobic conditions for 3 h and then underwent reoxygenation for 6 h in the normoxic incubator. The small interfering RNAs (siRNAs) specifically targeting lncRNA-XIST were purchased from Ribobio (China).

### 2.3. Luciferase Reporter Assay

The starBase database (https://starbase.sysu.edu.cn/) was utilized to predict the relationship between XIST and miR-217. Luciferase reporter assay was conducted to confirm the regulatory relationship. After amplifying the full length, XIST-WT vector was constructed, and XIST-MUT vector was constructed by point mutation kit (Hanbio Biotechnology Co., Ltd. Shanghai, China). XIST-WT or XIST-MUT was cotransfected into H9C2 cells with miR-217 mimic or miR-NC, respectively, by TurboFect. The luciferase activity was determined by kit after 48 hours using a dual luciferase reporter system (Promega).

### 2.4. RNA Isolation and Quantitative Real-Time PCR Analysis

Heart tissue samples (50-100 mg) were collected, and total RNA of cells was extracted by the TRIzol method. RNA was reverse-transcribed to cDNA according to the instructions of reverse transcription kit. qPCR reaction was carried out with All in-one miRNA kit (GeneCopoeia, USA), and the relative expression was calculated by the 2^-*ΔΔ*CT^ method. Primer sequences for PCR were shown in Table 1.

### 2.5. ELISA Assay

The contents of CK, CK-MB, LDH, and HBDH in serum were determined by ELISA kit (Beyotime, China).

### 2.6. Measurement of Myocardial Infarct Volume

Myocardial infarction area was detected by 1% TTC staining. After the hearts of rats in each group were frozen at -20°C for 30 min, they were cut into 5 slices of equal thickness, put into 1% TTC dye solution, dyed in 37°C constant temperature water bath for 30 min, washed and sliced with PBS for 3 times, and fixed with 10% formaldehyde for 12 h. Images were collected and processed by ImageJ software.

### 2.7. Assessment of ROS, SOD, LDH, and MDA

The ROS were determined by ROS Assay Kit (DCFH-DA, Sigma-Aldrich, Merck KGaA) and were incubated with DCFH-DA (10 mM/L) for 30 min at 37°C in the dark. The ROS level was detected by flow cytometry. The concentrations of SOD, LDH, and MDA in rat myocardium were detected by chemical colorimetry. LDH was detected by ELISA assay, SOD was detected by the xanthine oxidase method, and MDA was detected by the thiobarbituric acid method. GAH-Px was detected by the colorimetric method. The relevant kits were purchased from Nanjing Jiancheng Bioengineering Institute.

### 2.8. Western Blot

Heart tissue samples (50-100 mg) and cardiomyocytes samples were collected and were fully lysed with RIPA lysate (Cell Signaling Technology, USA) for 30 min, and the protein concentration was detected with BCA protein detection kit. After 10% SDS-PAGE gel electrophoresis, the protein was transferred to PVDF membrane, and 5% skimmed milk powder blocked the protein at room temperature for 2 hours. The specific primary antibody (Abcam, UK) was incubated with PVDF membrane overnight at 4°C, and the corresponding secondary antibody (Abcam, UK) was incubated with PVDF membrane at room temperature. Chemiluminescence was developed using a hypersensitive ECL kit (Baiaosi, China).

### 2.9. Flow Cytometry Assay

After washing with PBS, the cells were resuspended, and 500 *μ*L of binding buffer was added into suspension. 20 *μ*L of annexin V-fluorescein isothiocyanate (Annexin V-FITC) was added into suspension, and cells were incubated at room temperature for 10 min. 20 *μ*L of propidium iodide (PI) was added, and cells were incubated without light at room temperature for 5 min. FACScan flow cytometer (Becton, USA) was utilized for measurement.

### 2.10. Statistical Analysis

Statistical analysis was performed using SPSS 25.0 software. Mean between groups was compared with one-way ANOVA, and *P* < 0.05 was considered statistically significant.

## 3. Results

### 3.1. Emodin Attenuated Myocardial Ischemia-Reperfusion Injury by Inhibiting XIST

**Figure 1:**
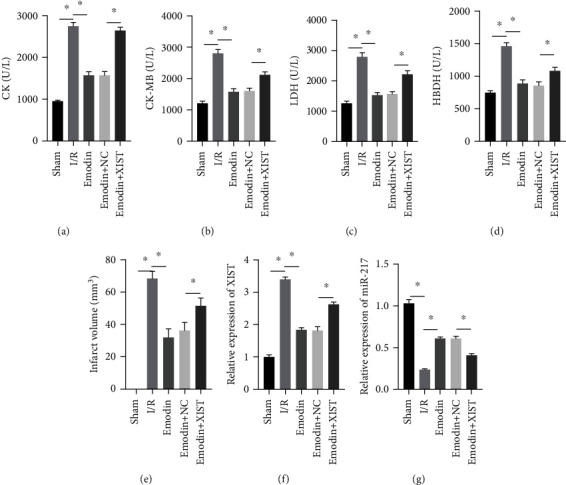
Emodin attenuated MIRI by inhibiting XIST. The contents of CK (a), CK-MB (b), LDH (c), and HBDH (d) in serum were determined by ELISA kit. Infarct volume (e) was detected via Image-Pro software. (e, g) The relative expression of XIST and miR-217 was evaluated by RT-qPCR. *n* = 16, ^∗^*P* < 0.05.

The activities of CK, CK-MB, LDH, and HNDH levels were increased in the I/R model group compared with the sham group, while the activities of CK, CK-MB, LDH, and HNDH levels were declined in the emodin group compared with the I/R model group (Figures 1(a)–1(d)). The activities of CK, CK-MB, LDH, and HNDH levels were increased in the emodin+XIST group compared with the emodin+NC group (Figures 1(a)–1(d)). Infarct size was obviously increased in the I/R model group compared with the sham group, while emodin declined the infarct size (Figure 1(e)). The relative expression of XIST was markedly raised in the I/R model group while decreased in the emodin group (Figure 1(f)). The relative expression of miR-217 was markedly decreased in the I/R model group while raised in the emodin group (Figure 1(g)).

### 3.2. Emodin Reduced Oxidative Stress by Inhibiting XIST

**Figure 2:**
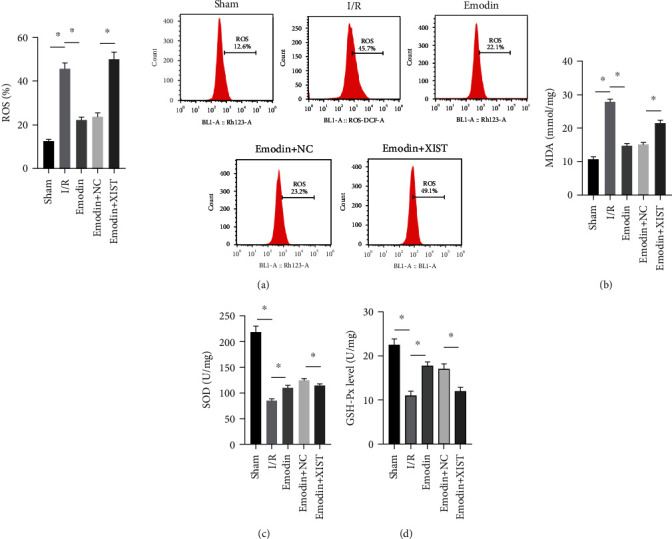
Emodin reduced oxidative stress by inhibiting XIST. (a) ROS was measured by flow cytometry. The contents of MDA (b), SOD (c), and GSH-Px (d) in myocardial tissues were determined. *n* = 16, ^∗^*P* < 0.05.

The ROS and MDA levels were increased in the I/R model group compared with the sham group, while the ROS and MDA levels were declined in the emodin group compared with the I/R model group (Figures 2(a) and 2(b)). In addition, the overexpression of XIST reversed the inhibitory effect of emodin on ROS and MDA levels. The activities of SOD were declined in the I/R model group compared with the sham group, while the activities of SOD were raised in the emodin group compared with the I/R model group (Figure 2(c)). The overexpression of XIST reversed the promoting effect of emodin on the activities of SOD (Figure 2(c)). The levels of GSH-Px were declined in the I/R model group compared with the sham group, while the levels of GSH-Px were raised in the emodin group compared with the I/R model group (Figure 2(d)). The overexpression of XIST reversed the promoting effect of emodin on the levels of GSH-Px.

### 3.3. Emodin Reduced Cardiomyocyte Apoptosis by Inhibiting XIST

**Figure 3:**
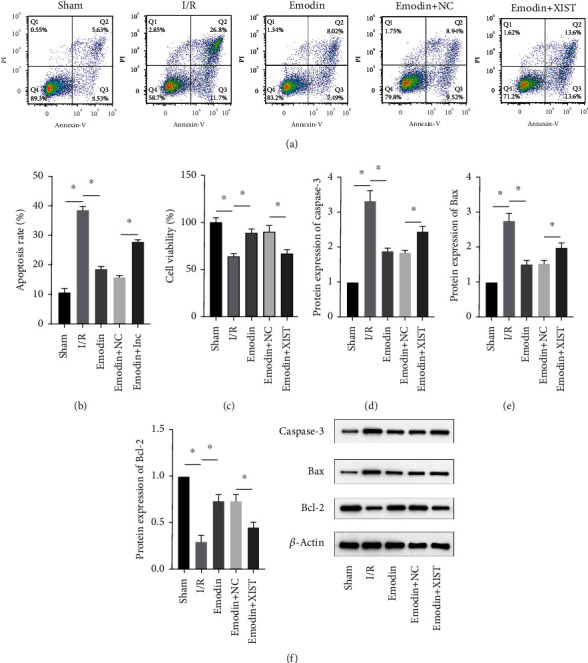
Emodin reduced cardiomyocyte apoptosis by inhibiting XIST. (a, b) Apoptosis was detected by flow cytometry. (c) CCK-8 assay was utilized to measure cell viability. (d–f) The protein expression of caspase-3 (d), Bax (e), and Bcl-2 (f) in myocardial tissues was determined. *n* = 3, ^∗^*P* < 0.05.

The cardiomyocyte apoptosis (Figures 3(a) and 3(b)) and the expression of caspase-3 (Figure 3(d)) and Bax (Figure 3(e)) were increased in the I/R model group compared with the sham group, while cardiomyocyte apoptosis and the expression of Bax and caspase-3 were declined in the emodin group compared with the I/R model group. In addition, the overexpression of XIST reversed the inhibitory effect of emodin on cardiomyocyte apoptosis and the expression of Bax and caspase-3. Cell viability was inhibited in the I/R model group compared with the sham group, while it was enhanced in the emodin group compared with the I/R model group. The overexpression of XIST reversed the promoting effect of emodin on cell viability (Figure 3(c)). The expression of Bcl-2 was decreased in the I/R model group compared with the sham group, while it was raised in the emodin group compared with the I/R model group (Figure 3(f)). The overexpression of XIST reversed the promoting effect of emodin on cardiomyocyte apoptosis and the expression of Bcl-2.

### 3.4. XIST Directly Regulated the Expression of miR-217

**Figure 4:**
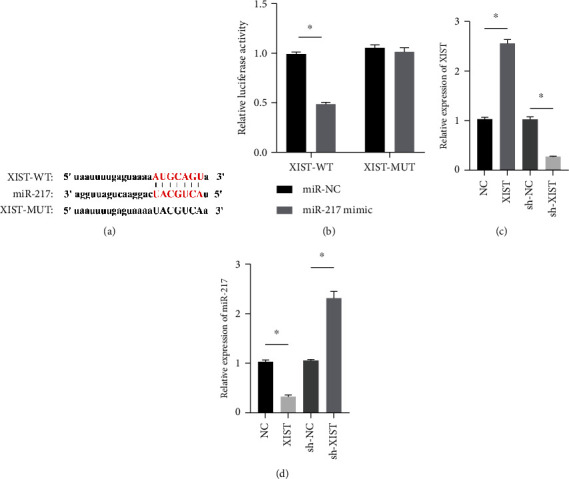
XIST directly regulated the expression of miR-217. (a) Predicted binding sites. (b) Inhibiting effect of miR-217 mimic on luciferase activity of XIST-WT. (c) The relative expression of XIST and miR-217 was evaluated by RT-qPCR (d). *n* = 3, ^∗^*P* < 0.05.

miR-217 was predicted to be regulated by XIST via TargetScan website (Figure 4(a)). The relative luciferase activity of XIST-WT was obviously decreased in miR-217 mimic while the relative luciferase activity of XIST-MUT was remained unchanged (Figure 4(b)). The XIST expression was observably raised in the XIST group and was markedly decreased in the sh-XIST group (Figure 4(c)). Overexpressed XIST obviously inhibited the miR-217 expression while sh-XIST markedly raised the miR-217 expression (Figure 4(d)).

### 3.5. Si-XIST Inhibited H/R-Induced Oxidative Damage of Cardiomyocytes via Inhibiting miR-217

**Figure 5:**
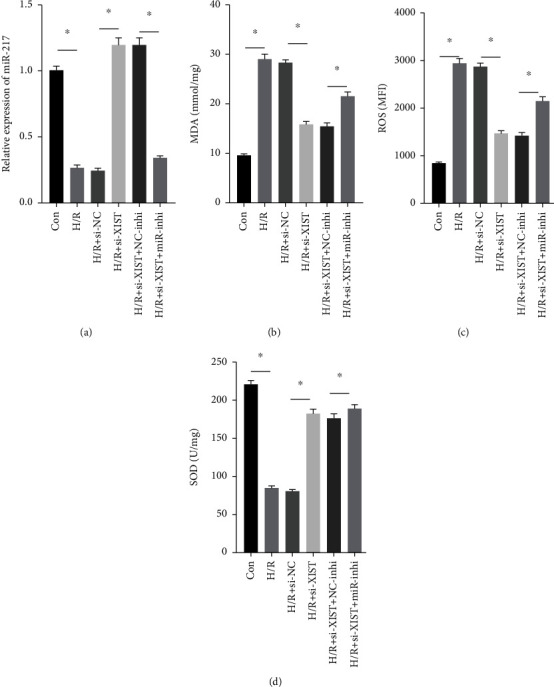
si-XIST inhibited H/R-induced oxidative damage of cardiomyocytes via inhibiting miR-217. (a) The relative expression of miR-217 was evaluated by RT-qPCR. The contents of MDA (b), ROS (c), and SOD (d) in myocardial tissues were determined. *n* = 16, ^∗^*P* < 0.05.

H/R treatment obviously decreased the miR-217 expression while si-XIST observably raised the miR-217 expression (Figure 5(a)). H/R treatment obviously raised ROS and MDA levels while si-XIST observably declined ROS and MDA levels, and miR-217 inhibitor markedly decreased ROS and MDA levels (Figures 5(b) and 5(c)). H/R treatment obviously inhibited the activities of SOD while si-XIST observably declined the activities of SOD, and miR-217 inhibitor markedly decreased ROS and MDA levels (Figure 5(d)).

### 3.6. miR-217 Directly Regulated the Expression of HDAC4

**Figure 6:**
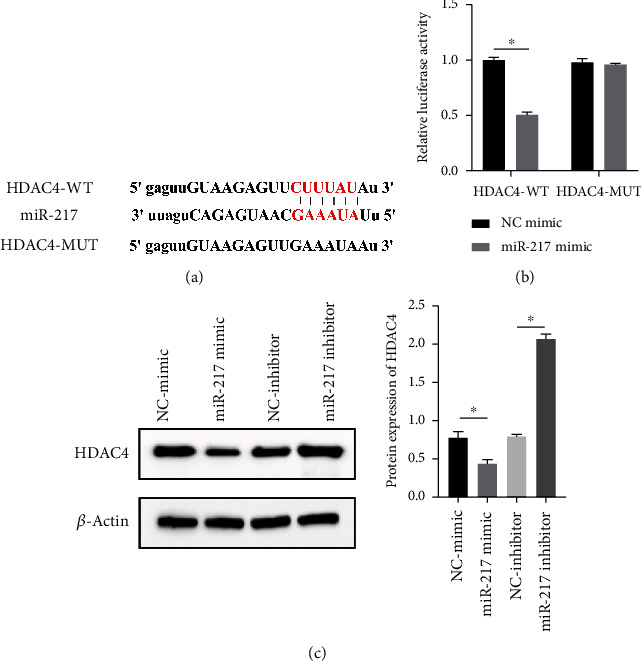
miR-217 directly regulated the expression of HDAC4. (a) Predicted binding sites. (b) Inhibiting effect of miR-217 mimic on luciferase activity of HDAC4-WT. (c) The protein expression of HDAC4 and miR-217 was evaluated by western blot. *n* = 3, ^∗^*P* < 0.05.

HDAC4 was predicted to be regulated by miR-217 via TargetScan website (Figure 6(a)). The relative luciferase activity of HDAC4-WT was obviously decreased in miR-217 mimic while the relative luciferase activity of HDAC4-MUT was remained unchanged (Figure 6(b)). The HDAC4 expression was observably raised in the miR-217 inhibitor group and was markedly decreased in the miR-217 mimic group (Figure 6(c)).

## 4. Discussion

Cardiovascular disease can cause reduced heart blood perfusion and insufficient oxygen supply to the heart, resulting in myocardial ischemia and hypoxia, abnormal cardiomyocyte metabolism, weak heart function, and threatening the life and health of patients [18]. The treatment for myocardial ischemia is blood flow reperfusion, which can restore cardiac oxygen supply and blood supply. However, studies reported that after ischemia and reperfusion, patients will have more serious arrhythmia, enlarged myocardial infarction, cardiac hypofunction, and even death, which is an obstacle in the clinical treatment of cardiovascular diseases [19].

The pathophysiological factors of MIRI include inflammatory stimulation, oxidative stress, cell necrosis, apoptosis, cardiomyocyte metabolism disorder, and other links, and MIRI is more closely related to reactive oxygen species free radicals and mitochondrial damage [20]. In MIRI, more oxygen free radicals form, and lipid peroxidation reacts with unsaturated fatty acids in membrane phospholipids, producing large amounts of toxic lipid peroxides, with impaired mitochondrial metabolism, and causing impaired cardiomyocyte structure [21]. Emodin can effectively prevent the expansion of inflammatory mediators and their biological effects and prevent serious complications mediated by inflammatory mediators. Present study demonstrated that the ROS and MDA levels were increased while the activities of SOD were decreased in the I/R model group. The ROS and MDA levels were decreased while the activities of SOD were raised in the emodin group. Therefore, emodin could improve the oxidative stress and cardiomyocyte apoptosis induced by MIRI, which is consistent with previous research results [22]. This study showed that ligation of the left anterior coronary artery in SD rats resulted in an increase in myocardial infarction area, while emodin decreased the myocardial infarction in MIRI model rats. In addition, emodin obviously decreased cardiac enzymes, oxidative stress, and apoptosis, which demonstrated that emodin exhibited protective functions against MIRI. This result is consistent with previous studies. Ye et al. had confirmed that emodin could alleviate MIRI [7]. Apoptosis may play a crucial role in the pathogenesis of myocardial ischemia-reperfusion injury. Relevant studies have confirmed that reducing the apoptosis of cardiomyocytes is beneficial to improve the prognosis of myocardial ischemia and reperfusion injury. Oxidative stress as well as the inflammatory response all play a crucial role in cardiomyocyte apoptosis.

Long chain noncoding RNA is a kind of RNA with a length of more than 200 nucleotides and does not encode proteins [23]. Although it does not have the ability to encode proteins, long chain noncoding RNA can regulate the expression level of target genes at the transcription and translation levels [24]. In MIRI, lncRNAs can regulate the activity of cardiomyocytes and the degree of reperfusion injury by targeting miRNA. Present study showed that the relative expression of XIST was markedly raised in the I/R model group while decreased in the emodin group, and the overexpression of XIST reversed the protective effect of emodin on myocardial infarction, oxidative stress, and cardiomyocyte apoptosis, which is similar to the results studied by Wang et al. [25]. lncRNAs can regulate the occurrence and development of heart disease and play a role by transcriptional or posttranscriptional control or competitive binding to miRNAs. XIST directly regulated the expression of miR-217, and XIST inhibited H/R-induced oxidative damage of cardiomyocytes via inhibiting miR-217.

In conclusion, emodin protected MIRI both *in vitro* and *in vivo* via inhibiting lncRNA XIST to upregulate miR-217. This study has some limitations. However, whether emodin can affect myocardial infarction area through lncRNA XIST/miR-217 needs further study. Because there are many regulatory pathways of myocardial injury, other regulatory pathways still need further research and evidence.

## Figures and Tables

**Table 1:** Primer sequence.

Target	Primer sequence (5′-3′)
miR-217	F: CGCAGATACTGCATCAGGAA
	R: CTGAAGGCAATGCATTAGGAACT

U6	F: CTCGCTTCGGCAGCACA
	R: AACGCTTCACGAATTTGCGT

XIST	F: GGTTCTGTCAAGATACTTTCCT
	R: CAATGAAGAGCTTGACGTG

*β*-Actin	F: ACGTCACGAACTACTAGCAAT
	R: TGTGTGCATGAGTCTCTCCACG

## Data Availability

Data to support the findings of this study is available on reasonable request from the corresponding author.
